# Quantitative evidence for the effects of multiple drivers on continental-scale amphibian declines

**DOI:** 10.1038/srep25625

**Published:** 2016-05-23

**Authors:** Evan H. Campbell Grant, David A. W. Miller, Benedikt R. Schmidt, Michael J. Adams, Staci M. Amburgey, Thierry Chambert, Sam S. Cruickshank, Robert N. Fisher, David M. Green, Blake R. Hossack, Pieter T. J. Johnson, Maxwell B. Joseph, Tracy A. G. Rittenhouse, Maureen E. Ryan, J. Hardin Waddle, Susan C. Walls, Larissa L. Bailey, Gary M. Fellers, Thomas A. Gorman, Andrew M. Ray, David S. Pilliod, Steven J. Price, Daniel Saenz, Walt Sadinski, Erin Muths

**Affiliations:** 1 U.S. Geological Survey Patuxent Wildlife Research Center, SO Conte Anadromous Fish Lab, 1 Migratory Way, Turners Falls, MA 01376, United States of America; 2 Department of Ecosystem Science and Management, Pennsylvania State University, University Park, PA, 16802, United States of America; 3 Department of Evolutionary Biology and Environmental Studies, University of Zurich, Switzerland; 4 KARCH, 2000 Neuchatel, Switzerland; 5 U.S. Geological Survey, Forest and Rangeland Ecosystem Science Center, Corvallis, OR, 97331, United States of America; 6 U.S. Geological Survey Patuxent Wildlife Research Center, Laurel, MD, 20708, United States of America; 7 U.S. Geological Survey, Western Ecological Research Center, San Diego, CA, 92101 United States of America; 8 Redpath Museum, McGill University, Montreal, Quebec, Canada; 9 U.S. Geological Survey, Northern Rocky Mountain Science Center, Aldo Leopold Wilderness Research Institute, Missoula, MT, 59801 United States of America; 10 University of Colorado, Boulder, Ecology and Evolutionary Biology Department, Boulder, CO, 80309 United States of America; 11 University of Connecticut, Department of Natural Resources and the Environment, Storrs, CT, 06269 United States of America; 12 School of Environment and Forest Sciences, University of Washington, Seattle, WA, 98195 United States of America; 13 U.S. Geological Survey, Wetland and Aquatic Research Center, Lafayette, LA, 70506 United States of America; 14 U.S. Geological Survey, Wetland and Aquatic Research Center, Gainesville, FL, 32653 United States of America; 15 Colorado State University, Department of Fish, Wildlife and Conservation Biology, Fort Collins, CO, 80523 United States of America; 16 U.S. Geological Survey, Western Ecological Research Center, Point Reyes Station, CA, 94956 United States of America; 17 Department of Fish and Wildlife Conservation, Virginia Tech, Blacksburg, VA, 24061 United States of America; 18 Greater Yellowstone Network Program, National Park Service, Bozeman, MT, 59715 United States of America; 19 U.S. Geological Survey, Forest and Rangeland Ecosystem Science Center, Boise, ID, 83702 United States of America; 20 Department of Forestry, University of Kentucky, Lexington, KY, 40506 United States of America; 21 Southern Research Station, Forest Service, U.S. Department of Agriculture, Nacogdoches, TX, 75961 United States of America; 22 U.S. Geological Survey, Upper Midwest Environmental Sciences Center, La Crosse, WI 54603, United States of America; 23 U.S. Geological Survey, Fort Collins Science Center, Fort Collins, CO, 80526, United States of America

## Abstract

Since amphibian declines were first proposed as a global phenomenon over a quarter century ago, the conservation community has made little progress in halting or reversing these trends. The early search for a “smoking gun” was replaced with the expectation that declines are caused by multiple drivers. While field observations and experiments have identified factors leading to increased local extinction risk, evidence for effects of these drivers is lacking at large spatial scales. Here, we use observations of 389 time-series of 83 species and complexes from 61 study areas across North America to test the effects of 4 of the major hypothesized drivers of declines. While we find that local amphibian populations are being lost from metapopulations at an average rate of 3.79% per year, these declines are not related to any particular threat at the continental scale; likewise the effect of each stressor is variable at regional scales. This result - that exposure to threats varies spatially, and populations vary in their response - provides little generality in the development of conservation strategies. Greater emphasis on local solutions to this globally shared phenomenon is needed.

The challenge in understanding the drivers of population dynamics, including species declines, is a classic example of the problem of pattern and scale in ecology^1^. On one hand, observations of ecological processes made at local scales often inadequately describe patterns at regional or continental scales^2^. In turn, conservation strategies derived from broad scale assessments are not always relevant to local conservation efforts. For over 20 years amphibian ecologists have generally followed the ‘declining population’ paradigm^3^, conducting research to identify the complex causes underlying declines^4^,^5^,^6^,^7^,^8^, and have called for the collection of additional data at more locations to further document and evaluate drivers of declines^5^,^6^,^7^,^9^. The implicit expectation is that observations from an increasing number of locations in space and time will help to identify universal laws underlying an observed phenomenon, from which more informed predictions and management decisions can be generated^10^,^11^. Information is treated equally in this passive approach to improving management, even though some insights are more valuable than others (i.e., those which can be taken advantage of to increase population size or distribution^10^). This has been a general criticism of conservation-oriented research and monitoring programs^12^. Since amphibian declines were first proposed as a global phenomenon over a quarter century ago, the collective efforts of thousands of scientists have helped quantify the severity of such declines and identified a handful of consensus causes^5^,^8^,^13^. These approaches have helped prioritize areas for conservation^14^,^15^, and while there have been a handful of local successes, the global decline was not halted^16^. In fact, declines have continued across North America since at least the 1960s^17^, with surprisingly few conservation success stories. This should not be unexpected – if the distribution of threats, population response, or causes of population declines vary among locations, then understanding causes of amphibian declines at large spatial scales may not reveal which threats are driving local populations^18^.

Existing large-scale analyses of amphibian declines have relied on expert opinion and range maps extrapolated from species’ detections to evaluate causes of endangerment^7^,^8^,^13^. For example, IUCN species assessments assume that threat categories reflect changes in population abundances or occurrence, though other criteria (including range size or population threat information) can result in the same level of species’ endangerment. A central finding has been that while dominant threats vary among large geographic areas, a significant proportion of declines have no identified causes, especially in North America^8^,^13^. Accordingly, a handful of threats including climate change, disease risk, and land use have been used to forecast continental-scale amphibian extinction risk^7^,^8^,^19^. However, these analyses and accompanying predictions are based on limited empirical data on amphibian metapopulations, and thus are largely untested. We use a continental scale, long-term data set of amphibian habitat occupancy from many species and populations to empirically test the generality of proposed drivers of decline. While previous assessments^8^,^13^ show that the distributions of drivers are not globally consistent, we test whether they are consistent among metapopulations, which is the appropriate scale for conservation action^20^. Specifically, we test whether populations are exposed to multiple threats, whether the intensity of exposure and population response to threats varies geographically, and whether, despite this variation, continental-scale patterns in declines can be identified.

## Results

**Figure 1:**
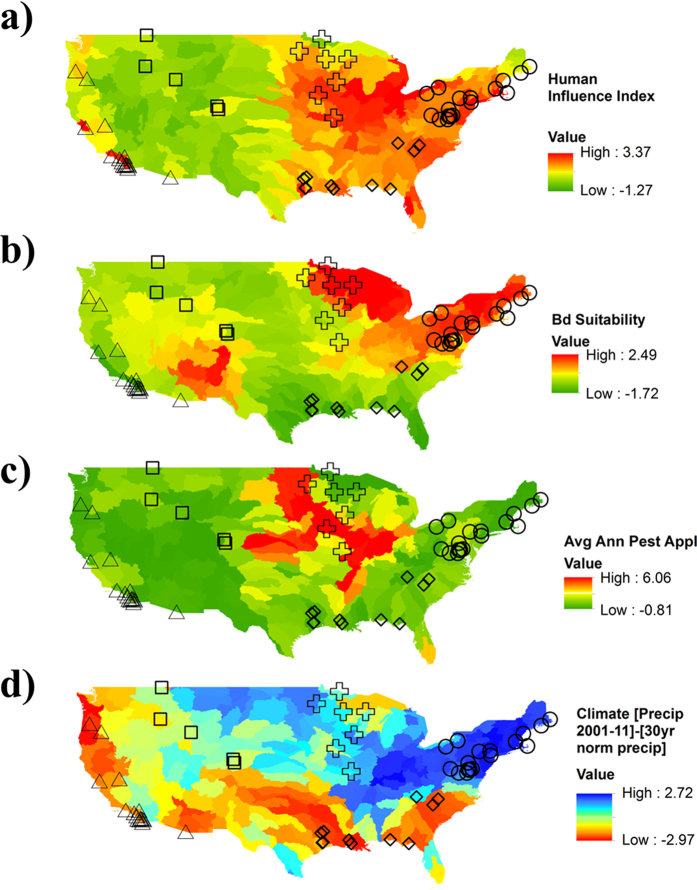
Spatial distribution of the four hypothesized threats to amphibian populations. Values are summarized and normalized at the HUC sub-basin scale, using ArcGIS (ver. 10.2. Redlands, CA: Environmental Systems Research Institute). (**a**) Human influence index. (**b**) Suitability for *Batrachochytrium dendrobatidis*. (**c**) Average annual pesticide application. (**a**–**c**) Red indicates higher than average threat intensity while green is lower than average. Locations of amphibian community data indicated with circles; symbols indicate region groupings (see Table 1; circles = Northeast, diamonds = Southeast, plus = Midwest, triangles = West coast, squares = Rocky Mountains). (**d**) The mean difference in the 30-year normal from the 2001–11 annual average precipitation; blue indicates wetter sub-basins with above average difference in precipitation while red indicates drier sub-basins.

**Figure 2:**
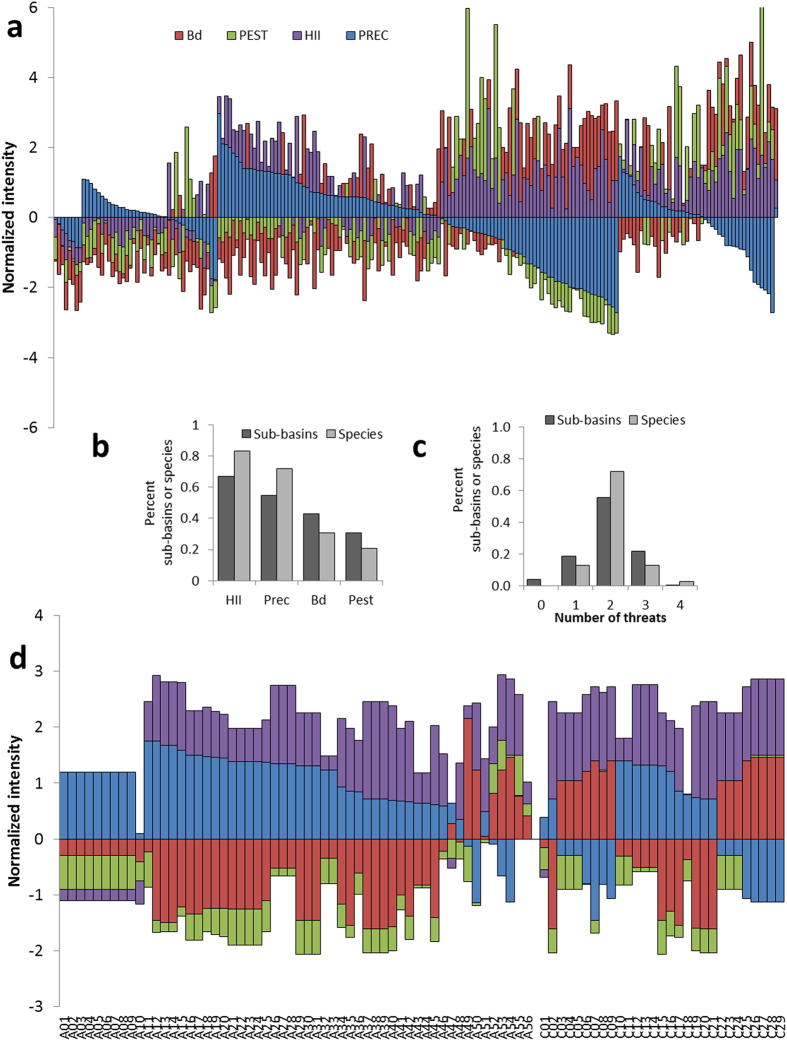
Normalized intensities for the 4 threats [HII = Human influence index; Bd = *Batrachochytrium dendrobatidis* suitability; Pest = annual average pesticide application; Prec = the difference in the 30-year normal from the annual average precipitation (2001–2011); droughts are represented as positive values to correspond with an increase in the threat] considered in our analysis (**a**) average exposure for each threat by sub-basin (sub-basins are sorted by number of threats with above-average values, and intensity of the climate threat; sub-basin identifiers omitted for clarity), (**b**) the frequency of above-average values of each of the four threats for all sub-basins and for those with time-series data for species, (**c**) the number of above-average threats for all sub-basins and species in our time-series, and (**d**) exposure to each threat for 54 anuran species and 2 anuran species complexes (A01–A56) and 29 caudate (C01–C29) species [corresponding species names in Table S3].

**Figure 3:**
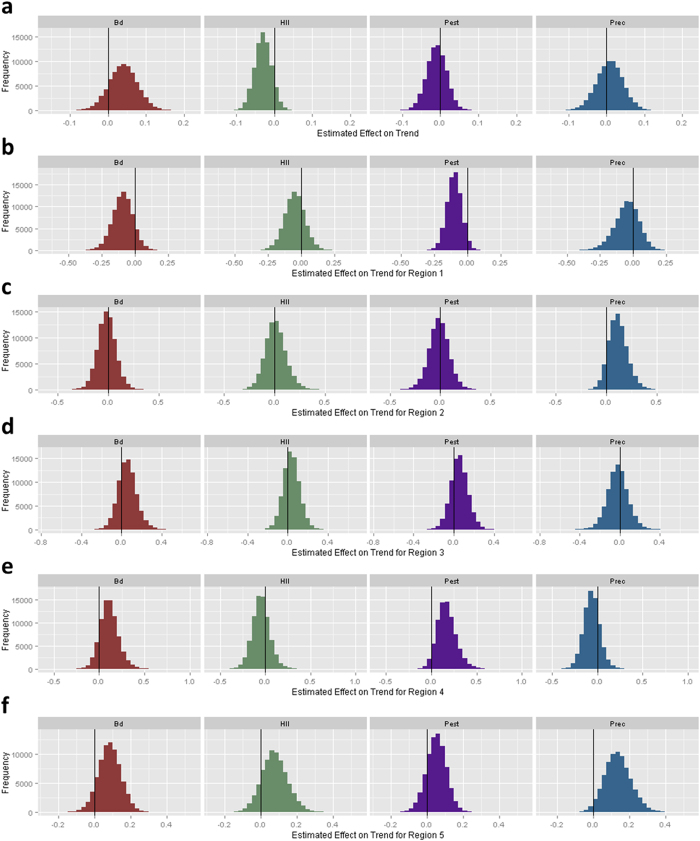
Posterior probabilities of estimated effects (β parameters) of the 4 threats on amphibian population trends for the national dataset (**a**) and for each region separately (**b**–**f**).

By relating spatially varying intensities of each threat (Fig. 1) to trends in the habitat occupancy of amphibian populations, we identified the relative contribution of each threat to decline in the number of populations across the continent. We provide strong evidence that the average rate of decline in amphibian metapopulations is 3.79% per year (95% CI {−2.49, −5.04}; Table 1), consistent with previous assessments^13^,^17^,^21^, and that similar rates of decline occur across all five regions we examined. This rate of decline means that on average, the number of occupied sites for an amphibian species will decrease by 50% within 19 years. However, we find not only that the number and relative intensity of threats varies geographically (Fig. 2), but also that population responses to threats vary when sub-basins were grouped into regions (Fig. 3). Because of this, our empirical data show that no single cause of decline can be identified. This results from substantial variation in the distribution and intensity of threats among sub-basins (Fig. 2a), which resulted in spatial variation in the number and combination of threat values to which amphibians in each sub-basin were exposed (Fig. 2b). High intensity of threats in sub-basins were most frequently land-use and drought (Fig. 2b) while high intensities of disease risk or pesticide application occurred in less than half of the sub-basins. Many sub-basins were exposed to multiple threats (Fig. 2c). Although the average annual population trend across all populations was negative (Table 1), no single threat was consistent in explaining the observed trends (Fig. 3a). Likewise, though we found that amphibian responses to these threats varied spatially, we did not find strong region-specific drivers of population trends (Fig. 3b–f). These results show that there is not a single consistent response to threats across the continental scale, and indicate that declines are discrete phenomena with spatially varying etiology.

The amphibian assemblages represented in our data differed among sub-basins, thus the exposure of each species to the number (Fig. 2b) and identity (Fig. 2c) of threats differs. Most species (72%) were exposed to higher-than-average intensities of two threats, with 13% exposed to three, and 13% exposed to a single threat. Two species (*Anaxyrus americanus* and *Pseudacris maculata*) were exposed to higher-than-average intensity levels of all four threats. On average, species were exposed most frequently to drought and Human Influence Index (HII) threats (72 and 83% of values, respectively, were greater than average). Most anurans were exposed to a combination of drought and human influence, while salamanders were most commonly exposed to a combination of human influence and *Bd*, or human influence and drought (Fig. 2d). Our findings suggest that in some populations, and for some species, a single factor may predominate, while in others a combination of threats drives population declines.

## Discussion

Our analysis uses data from across the United States to empirically test the relationship between change in the number of amphibian populations and hypothesized threats. We did not find support for a consistent relationship between rates of amphibian declines and distribution of stressors at the continental level. Instead our results suggest that declines result from locally driven processes and the influence of multiple interacting stressors that in turn lead to emergent declines at the continental scale. Our use of occupancy models provides statistically unbiased information that populations of amphibians are in decline across the United States. This result is more alarming than previous work identifying declining trends in abundance in North American amphibian populations^17^ because we show that populations themselves go locally extinct. The absence of a single cause of these declines, even at smaller regional scales, suggests that even regional conservation action plans may be ineffective in combatting threats to amphibian populations.

While there is value in empirically testing hypothesized factors underlying amphibian declines, the continuing pace of population extirpations and declines in abundance suggests a need for a new approach. Discussions at the 1st World Congress of Herpetology in Canterbury, UK, a quarter century ago, presaged the documentation of serious and global declines in amphibian populations. However, there is no longer a lack of research on potential causes of decline, as was the case in 1989. Rather, broad recognition of the multitude and complexity of factors driving amphibian declines^9^ emphasizes the need to increase the development and application of local science-based strategies for conservation of amphibian populations. In fact, understanding the causes of a decline may not necessarily lead to better management; for example, identifying populations which are at risk for Bd-induced decline has not led directly to better management, as few proposed solutions have been effective in the field^22^. More generally, understanding the causes of declines does not always mean that the best management action can be identified or implemented. While declines of amphibians and loss of biodiversity are globally consistent problems, most conservation actions must be implemented locally, at the scale of a population or metapopulations, to be most effective. Because we find that amphibian populations are exposed to multiple drivers and because the degree of exposure varies spatially, a single recipe for conservation is impractical. While some local conservation actions have proven successful^23^ thus far, solutions to threats at large scales are elusive^24^. Assuming few technological constraints (which may be addressed through research), a focus on local drivers could improve conservation. Similar to the principle of subsidiarity in solving water management problems^25^, amphibian conservation can beneficially consider global context, but focus on local actions.

Developing feasible strategies to increase population sizes, maintain population distributions, and support population resilience to environmental change requires two seemingly paradoxical capacities: a better understanding of which threats are most important locally, and a willingness to test conservation actions despite uncertainty in the identification of threats and the response of populations to management. We may use scientific approaches that allow us to learn about the causes of the declines while we implement conservation on the ground. This approach to developing management policies (e.g., under an adaptive management framework^26^), where we reduce uncertainties about the effect of specific management strategies on the target populations through active management, has proven successful (e.g., harvest management^27^). Under this approach to conservation, the investment in research is related directly to the potential improvement for populations under management^26^,^28^. Some uncertainty will always remain regardless of research, but the ability to take conservation actions given uncertainty is needed, especially where populations are facing catastrophic declines^29^. A shift in the approach to amphibian conservation towards action under uncertainty could improve efficiency in optimizing management in response to local population declines. This would include explicit identification of uncertainties which most matter to choosing among management options, greater transparency in management decision making, and increased sharing of successes and failures in a formal framework for evidence-based conservation decision making. This approach to learning – about what threatens local populations and what strategies will work to maintain populations – may be best implemented when resource managers incorporate more quantitative rigor into the development and application of management strategies, and when researchers make science more understandable, and immediately relevant, to managers.

## Experimental Procedures

We obtained unbiased estimation of trends in amphibian occurrence probabilities (i.e., changes in the number of populations that occur when local extinctions exceed (re-)colonizations within a metapopulation) from 61 study areas across North America (Fig. 1; Table S1), incorporating data from 389 time-series of 83 species of amphibians (Table S2) using a hierarchical occupancy model^30^. Study areas were mainly Federal and State protected areas, and we had no *a priori* expectation that amphibian populations or resource conditions were in decline or deteriorating when surveys were initiated. Data came from field surveys of multiple habitat units (e.g., wetlands, stream segments, forest patches) within each protected area and included habitats where amphibian species were expected to have a chance of occurring. Site selection was unconditional on initial amphibian presence, and sites at each study area were selected under a probabilistic design (e.g., simple random sampling, stratified random sampling); this means that though site selection differed among study areas, statistical inference is appropriate at the scale of each study area. Survey methods differed among habitats and species, but were generally chosen to maximize the probability of detecting the target species and sometimes multiple standard methods for detecting amphibians were used. Multiple species may have been detected during a single survey occasion. Surveys were repeated on multiple occasions within a time period when each species had the possibility of occupying a site (i.e., sites were assumed to be demographically ‘closed’ during the duration of surveys). These detection-nondetection data were analyzed in our occupancy model, and used to estimate detection probabilities, thereby accounting for unequal effort and other sources of observation error so that inference is on the (true) state of a population (i.e., present or absent) and is unconditional on whether or not a species was detected at a site during sampling occasions^31^.

### Statistical model

Using Markov Chain Monte Carlo methods^30^,^32^, we estimated trends of amphibian populations for each metapopulation (i.e., each combination of species and study area), and the effects of four of the most widely cited threats [land-use, contaminants, climate change and disease risk^5^,^6^,^8^,^13^], as there is evidence that these threats have contributed to the extinction of local populations [e.g., (26–29)]. Trends were estimated for each metapopulation, and represent the balance of local extinctions and colonizations over each time-series; a negative trend occurs when extinctions exceed colonizations. We defined 5 regions as geographic clusters of protected areas.

For all studies, multiple observations were collected within a year, which allowed us to estimate unbiased trends across years. We denote year by *y* and time-series by *k*, so that ψ_*yk*_ is the probability a site in the *k*^th^ time-series in the *y*^th^ year is occupied by a given species and *p*_*yk*_ is the probability of observing a given species during a visit to a site, given that site is occupied by the species. We denote the number of visits to the *i*^th^ site in a given year for a given time series as N_*iyk*_ and the number of times the species was observed at the site during those visits as Y_*iyk*_. The true, but unobserved, state of a site is denoted by z_*iyk*_, where z = 1 if a site is occupied and z = 0 if not.

Observations Y_*iyk*_ are modeled with a binomial distribution as:


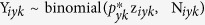



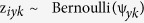


Note the binomial probability is conditional on the true occupancy state of the site, which is a Bernoulli trial with probability of occurrence for the specific combination of year and time-series. To accommodate variation of detection probabilities among different years and time-series, we assumed a random distribution for the detection parameters:


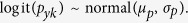


We estimated occupancy trends as a derived parameter within the MCMC algorithm, calculating the average rate of change in number of occupied sites (λ) in each time-series as a function of the estimated trend parameters in the logit-linear model as


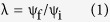


where ψ_i_ and ψ_f_ are occupancy at the start and end of each time series.

Our interest was in understanding the factors associated with annual variation in occupancy probabilities. We expected that relationships would not be evident among threats and continental-scale population trends if declines are driven by different factors among populations, but that relationships may be detectable at regional scales if these threats influence populations more consistently at a smaller spatial scale.

We fit a generalized linear mixed model, where the model included two random components, α_k_, a random-effect for time-series intercept, and δ_yk_, a random-error component to account for sub-sampling of sites within each combination of year and time-series, where:


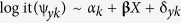


Distributions for random-effects were specified as:


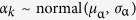



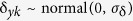


A fixed model, **βX**, was used to specify the effect of year (continuous), threat, and interaction between threat and year. Covariates for threats were scaled and centered to have a mean of 0 and standard deviation of 1 and year was centered to have a mean of 0 at the level of the time-series. To account for model uncertainty in the inclusion of parameters and minimize potential effects of correlation among threat covariates, we fit parameters using a Bayesian LASSO (least absolute shrinkage and selection operator) approach^33^,^34^, by specifying the **β** parameters to come from a double-exponential distribution, where:


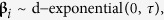


and τ, the regularization parameter was estimated from the data used to fit the model (example code to implement a LASSO provided in Supporting Online Material). To allow for a single value of τ to be estimated, we specified year covariates and interactions between year and threats to have a mean of 0 and standard deviation of 1 when fitting the model. We then back-transformed estimates so the estimated effect of year represented the average annual change in occupancy across all time-series and the estimated interaction effects represented the average annual effect of a 1-standard deviation increase in the value of a threat. The Markov Chain Monte Carlo-estimated posterior probabilities of the parameters (i.e., the standardized partial regression coefficients) can be interpreted as the relative contribution of each threat to the observed population trends.

Priors were chosen as follows: logit^−1^(*μ*_α_) and logit^−1^(*μ*_p_) were uniform (0,1) on the real scale; *σ*_p_, *σ*_α_, and *σ*_δ_ were uniform (0, 3); and τ was uniform (0, 20). Models were fit using JAGS run via R (v.2.15.2; R Core Development Team 2012). We used a burn-in sample of 5000 iterations and then estimated the posterior distribution of parameters based on the next 25000 samples. Three chains were run and model convergence was assessed based on Gelman-Rubin statistics^35^.

### Threats

Disease risk was defined as the suitability for occurrence of the fungal pathogen *B. dendrobatidis* (Bd)^36^, as it is classified as one of the “enigmatic” causes of amphibian decline^13^,^37^. We used spatially referenced predictions of Bd suitability for the United States, which were generated under the environmental niche model described in^36^ (provided by D. Olsen, US Forest Service). In a binomial regression model, the detection of *Bd* was found to be different among major ecosystems of the US, generally increasing with number of species, and increasing with decreasing minimum temperature (details of model found in^36^). Predicted suitability under this model is qualitatively similar to the global model^38^ in^8^. There are no comparable data on other emerging amphibian pathogens such as *Ranavirus* or *Batrachochytium salamandrivorans*.

The threat from climate change relates to an increased risk of extinction in response to changes in precipitation, mainly resulting in increased frequency and intensity of droughts^39^. This threat from climate change was calculated as the mean difference in mean annual precipitation from (2001–2011) and the 30-year normal (1981–2010; available from: http://www.prism.oregonstate.edu/, accessed 13 March 2014), which was a proxy for water availability. To obtain the difference in annual average precipitation during the period of our surveys (2001–11), we subtracted the 10-year average annual precipitation for each sub-basin over the period of record (monthly data obtained from PRISM for 2001–11; http://www.prism.oregonstate.edu/recent/; accessed 13 March 2014) from the 30-year (1981–2010) normal precipitation (http://www.prism.oregonstate.edu/normals/; accessed 13 March 2014).

The threat from land use change was evaluated because it is the single most important threat to biodiversity in general^40^, and both human influence (Human Influence Index; HII^41^) and the application of pesticides^42^,^43^ are components of this threat. The human influence index (HII), developed by the Wildlife Conservation Society and the Center for International Earth Science Information Network^41^, is an index of direct human disturbance which incorporates human population density, land transformation, accessibility, and electrical power infrastructure to represent direct human influence on the landscape. We summarized the 1-km^2^-pixel HII raster map provided by^41^ as the mean HII within each sub-basin.

The threat from contaminants was described by the estimated annual pesticide use values for compounds known or hypothesized to be important to amphibians (Table in^44^ and updated by K. Smalling, USGS pers. comm.). We used the ‘EPest-high’ application estimation method from^42^,^43^ for each county from 2001–11, which treats non-reporting of application as missing values which are estimated from nearby data. Values were summarized at the sub-basin scale by weighting the average application by the area of the county falling within each sub-basin, and thus reflect the average pesticide application per unit area.

For each threat, we calculated the average, normalized intensity (the value of each threat) within each sub-basin (i.e., United States Geological Survey (USGS) HUC4-scale sub-basins; http://pubs.usgs.gov/wsp/wsp2294/html/pdf.html) for use in our occupancy models. We investigated the correlations among the normalized threats for all 204 sub-basins in the continental United States using the Band Collection Statistics tool in Spatial Analyst in ArcGIS 10.2. Not all sub-basins had amphibian population data; correlations among threats for the subset of sub-basins (n = 55) with amphibian species data were determined independently. All correlations were <0.47, except a positive correlation between Bd and climate for the 55 sub-basins with amphibian data (ρ = 0.648) results from the inclusion of precipitation in the suitability model of^36^.

## Additional Information

**How to cite this article**: Grant, E. H. C. *et al*. Quantitative evidence for the effects of multiple drivers on continental-scale amphibian declines. *Sci. Rep.* **6**, 25625; doi: 10.1038/srep25625 (2016).

## Supplementary Material

Supplementary Information

## Figures and Tables

**Table 1:** Estimated annual trends (transformed from the estimated of and lower and upper limits of 95% credible intervals) in number of occupied sites, across all sites and by region.

	**mean annual trend**	**2.5%**	**97.5%**
All sites	−3.79%	−2.49%	−5.04%
Region 1 (Northeast)	−3.57%	−1.87%	−5.21%
Region 2 (Midwest)	−3.15%	−0.41%	−5.75%
Region 3 (Southeast)	−2.30%	−0.81%	−3.70%
Region 4 (West coast)	−5.17%	−3.00%	−7.24%
Region 5 (Rocky Mountains)	−4.93%	−2.37%	−7.43%
