# Supplementary material for: Quantitative evidence for the effects of multiple drivers on continental-scale amphibian declines
Source: Sci Rep. 2016 May 23;6:25625. doi: 10.1038/srep25625 (PMC4876446; doi:10.1038/srep25625)
Supplement: Supplementary Information [file srep25625-s1.pdf]

Supporting online material for article: Quantitative evidence for the effects of multiple drivers on continental-scale amphibian declines

Authors: Evan H Campbell Grant, David AW Miller, Benedikt R Schmidt, Michael J Adams, Staci M Amburgey, Thierry Chambert, Sam S Cruickshank, Robert N Fisher, David M Green, Blake R. Hossack, Pieter TJ Johnson, Maxwell B Joseph, Tracy Rittenhouse, Maureen Ryan, J Hardin Waddle, Susan C Walls, Larissa L Bailey, Gary M. Fellers, Thomas A Gorman, Andrew M Ray, David S Pilliod, Steven J Price, Dan Saenz, Erin Muths

Table S1. Study areas from which amphibian survey data collected; <MetadataID> is a unique identifier; <Area Name> is the name of the study area; <Land Owners> are the agencies responsible for management of the study area; <Lat> and <Lon> are the Latitude and Longitude of the center of the study area; <Habitat type> indicates whether the data come from surveys of wetlands, streams or terrestrial sites; <Number of Sites> is the total number of habitats for a given study area; <Number of Years> is the length of the time series.

MetadataID	Area Name	Land Owners	Lat	Lon	Habitat type	Number of Sites	Number of Years
1000	Colorado ponds	NPS, private	39.82594	-105.584	wetland	213	5
1111	St Marks NWR	US Fish and Wildlife Service	30.02816	-84.4334	wetland	26	5
2000	California ponds	NPS, private	37.61076	-121.943	wetland	50	5
2222	St Marks NWR	US Fish and Wildlife Service	30.02867	-84.4264	wetland	60	5
3000	Daniel Boone Conservation Area (DBCA)	Missouri Department of Conservation	38.77624	-91.3925	wetland	51	8
3333	Santa Monica Mountains and Simi Hills (in or near Santa Monica Mountains National Recreation Area)	National Park Service, CA State Parks, Mountains Recreation and Conservation Authority, Mountains Restoration Trust, Conejo Open Space Conservation Authority,	34.11808	-118.766	stream	74	13
4000	Great Smoky Mountain National Park	National Park Service	35.68452	-83.5019	terrestrial	101	4
4444	Eglin AFB A. bishopi monitoring	U.S. Government (Eglin AFB)	30.49806	-86.6859	wetland	109	10
6000	Davy Crockett National Forest	USFS	31.29879	-95.1015	wetland	4	14
6100	Stephen F. Austin Experimental Forest	USFS	31.49261	-94.7718	wetland	4	14
6300	Willamette Valley Mid-level Monitoring	USFWS, BLM, City of Eugene	44.56866	-123.171	wetland	38	10
6870	C&O NHP Lentic	NPS	38.93	-77.1	wetland	65	8
7000	Broad River		34.71779	-81.5098	stream	42	4
7020	C&O NHP Lotic	NPS	38.99	-77.24	stream	7	8
7030	Atchafalaya Basin- TruckSites	USFWS, Louisiana Department of Wildlife and Fisheries, U.S. Army Corps of Engineers	30.45518	-91.7017	wetland	34	6
7031	Atchafalaya Basin- BoatSites	Louisiana Department of Wildlife and Fisheries, Louisiana State Lands	30.02434	-91.43	wetland	30	6
7050	Upper Mississippi River National Wildlife and Fish Refuge	Fish Wildlife Service	43.21833	-91.068	wetland	59	4
7060	St. Croix National Scenic Riverway	National Park Service	46.01443	-92.3629	wetland	65	4
7070	Voyageurs National Park	National Park Service	48.48322	-92.8363	wetland	56	4
7100	Charlotte, NC Stream Salamanders	Mecklenburg County Nature Preserves, Kay Killian Conservation Easement, Ramah Creek Conservation Easement, Davidson College	35.42154	-116.882	stream	19	5
7280	Yosemite National Park Mid-Level Monitoring Area	NPS	37.88196	-119.534	wetland	172	6
7460	Southwest Arizona	Fish and Wildlife Service	31.5	-111.5	wetland	41	7

7550	Monitoring the status of the Oregon spotted frog	USFS, BLM, Sunriver Nature Center	43.61231	-121.764	wetland	93	4
7600	Acadia National Park	NPS	44.33	-68.366	wetland	28	8
7610	Cape Cod National Seashore	NPS	41.85	-70.06	wetland	30	7
7620	Patuxent Research Refuge	USFWS	39.08	-76.77	wetland	100	9
7630	Eastern Massachusetts NWR Complex	USFWS	42.39	-71.47	wetland	39	8
7640	Gettysburg National Military Park	NPS	39.8	-77.24	wetland	20	4
7650	Rachel Carson NWR	USFWS	43.36	-70.55	wetland	12	8
7660	Canaan Valley NWR & State Park	USFWS, WV Dept of Natural Resources	39.08	-79.38	wetland	109	8
7670	Erie NWR	USFWS	41.77	-79.95	wetland	18	8
7680	Moosehorn NWR	USFWS	45.08	-67.31	wetland	43	6
7690	Great Swamp NWR	USFWS	40.71	-74.5	wetland	95	8
7700	Iroquois NWR	USFWS	42.5	-78.5	wetland	20	8
7710	Rock Creek Lotic	NPS	38.97	-77.044	stream	5	8
7720	Prince William Lotic	NPS	38.58	-77.38	stream	19	4
7740	Trout Lake	Wisconsin Department of Natural Resources	46.0306	-89.654	wetland	10	5
7750	Tamarac National Wildlife Refuge	National Parks Service	46.98179	-95.618	wetland	10	4
7760	St. Croix National Scenic Riverway	National Parks Service	46.01443	-92.3629	wetland	10	5
7770	Upper Mississippi River National Wildlife and Fish Refuge	Fish and Wildlife Service	43.21833	-91.068	wetland	5	5
7800	Glacier National Park	NPS	48.95	-114.43	wetland	1098	8
7811	Big Thicket National Preserve South	NPS	30.14	-94.92	wetland	32	3
7812	Big Thicket National Preserve North	NPS	30.17	-94.85	wetland	20	2
7840	MCBCP Arroyo Toad Surveying	U.S. Government (Camp Pendleton Marine Corps Base)	33.3795	-117.447	stream	335	11
7850	San Diego River post-fire	US Forest Service, City of San Diego	33.02453	-116.718	stream	30	4
7860	San Dieguito River post-fire	CA Dept. of Fish and Wildlife, City of San Diego, County of San Diego, San Dieguito River Park JPA, US Forest Service	33.08866	-116.909	stream	54	4
7870	Sweetwater River post-fire	CA State Parks	32.89892	-116.586	stream	27	5
7880	Tijuana River post-fire	City of San Diego	32.58535	-116.747	stream	13	4
7900	Yellowstone and Grand Teton National Park	NPS	43.5	-110.85	wetland	1113	10
7920	Neal Smith	Fish Wildlife Service	41.56339	-93.2796	wetland	17	3
8100	Rocky Mountain National Park	NPS	40.23	-105.77	wetland	332	10
8101	Bighorn Crags, Salmon River Mountains, Idaho	USFS, Salmon-Challis National Forest	45.12367	-114.583	wetland	79	20
8170	Shenandoah National Park	NPS	38.59	-78.371	terrestrial	260	6
8180	Rock Creek Park	NPS	38.97	-77.044	wetland	10	7
8190	Delaware Water Gap NRA	NPS	41.18	-75.9	wetland	45	2
8200	Walkill River NWR	USFWS	41.24	-74.56	wetland	26	3
9370	Elliott Chaparral Preserve (ELL)	Nature Reserve System of the University of California	32.89221	-117.095	terrestrial	10	15
9380	San Diego Zoo's Wild Animal Park (WAP)	City of San Diego	33.09522	-116.981	terrestrial	20	18
9385	Southern California Mountains	USDA, Forest Service	34.0495	-117.15	stream	221	9

9390	California pitfall trapping	Irvine Ranch Conservancy/The Nature Conservancy, BLM, National Park Service, California State Parks	32.95569	-117.302	terrestrial	117	17
9395	Orange County pitfalls	Puente Hills Landfill Native Habitat Preservation Authority, Irvine Ranch Conservancy, The Nature Conservancy, Audobon California	33.76742	-117.765	terrestrial	79	9

Tbale S2. Amphibian genus and species from each study area (referenced by the MetadataID; see Appendix 1 for study area name and location), and the HUC4 subwatershed code.

MetadataID	HUC4	Genus	Species
1000	1019	Anaxyrus	boreas
1000	1019	Ambystoma	mavortium
1000	1019	Anaxyrus	woodhousii
1000	1019	Lithobates	catesbeianus
1000	1019	Lithobates	pipiens
1000	1019	Pseudacris	maculata
1000	1405	Anaxyrus	boreas
1000	1405	Ambystoma	mavortium
1000	1405	Anaxyrus	woodhousii
1000	1405	Lithobates	catesbeianus
1000	1405	Lithobates	pipiens
1000	1405	Pseudacris	maculata
1000	1301	Anaxyrus	boreas
1000	1301	Ambystoma	mavortium
1000	1301	Anaxyrus	woodhousii
1000	1301	Lithobates	catesbeianus
1000	1301	Lithobates	pipiens
1000	1301	Pseudacris	maculata
1111	312	Pseudacris	crucifer
1111	312	Pseudacris	nigrita
1111	312	Pseudacris	ocularis
1111	312	Pseudacris	ornata
2000	1806	Anaxyrus	boreas
2000	1806	Ambystoma	californiense
2000	1806	Lithobates	catesbeianus
2000	1806	Pseudacris	regilla
2000	1806	Rana	draytonii
2000	1806	Taricha	torosa
2000	1805	Anaxyrus	boreas
2000	1805	Ambystoma	californiense
2000	1805	Lithobates	catesbeianus
2000	1805	Pseudacris	regilla
2000	1805	Rana	draytonii
2000	1805	Taricha	torosa
2222	312	Ambystoma	talpoideum
3000	1030	Lithobates	sylvaticus
3333	1807	Anaxyrus	boreas
3333	1807	Pseudacris	cadaverina

3333	1807	Pseudacris	regilla
3333	1807	Taricha	torosa
4000	601	Desmognathus	fuscus
4000	601	Desmognathus	imitator
4000	601	Desmognathus	wrighti
4000	601	Eurycea	wilderae
4000	601	Plethodon	glutinosus (complex)
4000	601	Plethodon	jordani
4000	601	Plethodon	serratus
4444	314	Ambystoma	bishopi
6000	1202	Acris	crepitans
6000	1202	Anaxyrus	woodhousii
6000	1202	Gastrophryne	carolinensis
6000	1202	Hyla	chrysoscelis
6000	1202	Hyla	versicolor
6000	1202	Incilius	nebulifer
6000	1202	Lithobates	catesbeianus
6000	1202	Lithobates	clamitans
6000	1202	Lithobates	palustris
6000	1202	Lithobates	sphenocephalus
6000	1202	Pseudacris	crucifer
6000	1202	Pseudacris	triseriata
6100	1202	Acris	crepitans
6100	1202	Anaxyrus	woodhousii
6100	1202	Gastrophryne	carolinensis
6100	1202	Hyla	chrysoscelis
6100	1202	Hyla	versicolor
6100	1202	Incilius	nebulifer
6100	1202	Lithobates	catesbeianus
6100	1202	Lithobates	clamitans
6100	1202	Lithobates	palustris
6100	1202	Lithobates	sphenocephalus
6100	1202	Pseudacris	crucifer
6100	1202	Pseudacris	triseriata
6300	1709	Ambystoma	gracile
6300	1709	Ambystoma	macrodactylum
6300	1709	Pseudacris	regilla
6300	1709	Rana	aurora
6300	1709	Taricha	granulosa
6870	207	Ambystoma	maculatum
6870	207	Anaxyrus	fowleri/americanus
6870	207	Ambystoma	opacum
6870	207	Hemidactylium	scutatum

6870	207	Hyla	versicolor/chrysoscelis
6870	207	Lithobates	catesbeianus
6870	207	Lithobates	clamitans
6870	207	Lithobates	palustris
6870	207	Lithobates	sphenocephalus
6870	207	Lithobates	sylvaticus
6870	207	Notophthalmus	viridescens
6870	207	Pseudacris	crucifer
7000	305	Anaxyrus	americanus
7000	305	Acris	crepitans
7000	305	Anaxyrus	fowleri
7000	305	Anaxyrus	terrestris
7000	305	Gastrophryne	carolinensis
7000	305	Hyla	chrysoscelis
7000	305	Hyla	cinerea
7000	305	Lithobates	catesbeianus
7000	305	Lithobates	clamitans
7000	305	Lithobates	palustris
7000	305	Lithobates	sphenocephalus
7000	305	Pseudacris	crucifer
7000	305	Pseudacris	feriarum
7000	305	Acris	gryllus
7020	207	Desmognathus	fuscus
7020	207	Eurycea	bislineata
7020	207	Pseudotriton	ruber
7020	207	Desmognathus	fuscus
7020	207	Eurycea	bislineata
7020	207	Pseudotriton	ruber
7030	808	Acris	blanchardi
7030	808	Anaxyrus	fowleri
7030	808	Gastrophryne	carolinensis
7030	808	Hyla	chrysoscelis
7030	808	Hyla	cinerea
7030	808	Hyla	squirella
7030	808	Incilius	nebulifer
7030	808	Lithobates	catesbeianus
7030	808	Lithobates	clamitans
7030	808	Lithobates	sphenocephalus
7030	808	Pseudacris	crucifer
7030	808	Pseudacris	fouquettei
7031	808	Acris	blanchardi
7031	808	Anaxyrus	fowleri
7031	808	Gastrophryne	carolinensis

7031	808	Hyla	chrysoscelis
7031	808	Hyla	cinerea
7031	808	Hyla	squirella
7031	808	Incilius	nebulifer
7031	808	Lithobates	catesbeianus
7031	808	Lithobates	clamitans
7031	808	Lithobates	grylio
7031	808	Lithobates	sphenocephalus
7031	808	Pseudacris	crucifer
7031	808	Pseudacris	fouquettei
7031	807	Acris	blanchardi
7031	807	Anaxyrus	fowleri
7031	807	Gastrophryne	carolinensis
7031	807	Hyla	chrysoscelis
7031	807	Hyla	cinerea
7031	807	Hyla	squirella
7031	807	Incilius	nebulifer
7031	807	Lithobates	catesbeianus
7031	807	Lithobates	clamitans
7031	807	Lithobates	grylio
7031	807	Lithobates	sphenocephalus
7031	807	Pseudacris	crucifer
7031	807	Pseudacris	fouquettei
7050	706	Anaxyrus	americanus
7050	706	Hyla	versicolor/chrysoscelis
7050	706	Lithobates	catesbeianus
7050	706	Lithobates	clamitans
7050	706	Lithobates	pipiens
7050	706	Pseudacris	crucifer
7050	706	Pseudacris	maculata
7050	706	Lithobates	sylvaticus
7050	708	Anaxyrus	americanus
7050	708	Hyla	versicolor/chrysoscelis
7050	708	Lithobates	catesbeianus
7050	708	Lithobates	clamitans
7050	708	Lithobates	pipiens
7050	708	Pseudacris	crucifer
7050	708	Pseudacris	maculata
7050	708	Lithobates	sylvaticus
7050	704	Anaxyrus	americanus
7050	704	Hyla	versicolor/chrysoscelis
7050	704	Lithobates	catesbeianus
7050	704	Lithobates	clamitans

7050	704	Lithobates	pipiens
7050	704	Lithobates	sylvaticus
7050	704	Pseudacris	crucifer
7050	704	Pseudacris	maculata
7060	703	Anaxyrus	americanus
7060	703	Hyla	versicolor/chrysoscelis
7060	703	Lithobates	clamitans
7060	703	Lithobates	pipiens
7060	703	Lithobates	septentrionalis
7060	703	Pseudacris	crucifer
7060	703	Pseudacris	maculata
7060	703	Lithobates	sylvaticus
7070	903	Anaxyrus	americanus
7070	903	Hyla	versicolor/chrysoscelis
7070	903	Lithobates	sylvaticus
7070	903	Pseudacris	crucifer
7070	903	Pseudacris	maculata
7070	903	Lithobates	clamitans
7070	903	Lithobates	pipiens
7070	903	Lithobates	septentrionalis
7100	305	Desmognathus	fuscus
7100	305	Eurycea	cirrigera
7100	305	Eurycea	guttolineata
7100	305	Gyrinophilus	porphyriticus
7100	305	Pseudotriton	ruber
7100	304	Desmognathus	fuscus
7100	304	Eurycea	cirrigera
7100	304	Eurycea	guttolineata
7100	304	Gyrinophilus	porphyriticus
7100	304	Pseudotriton	ruber
7280	1804	Anaxyrus	canorus
7280	1804	Pseudacris	regilla
7280	1804	Rana	sierrae
7460	1505	Anaxyrus	cognatus
7460	1505	Ambystoma	mavortium
7460	1505	Anaxyrus	punctatus
7460	1505	Gastrophryne	olivacea
7460	1505	Hyla	arenicolor
7460	1505	Incilius	alvarius
7460	1505	Lithobates	catesbeianus
7460	1505	Lithobates	chiricahuensis
7460	1505	Scaphiopus	couchii
7460	1505	Smilisca	fodiens

7460	1505	Spea	multiplicata
7460	1508	Anaxyrus	cognatus
7460	1508	Ambystoma	mavortium
7460	1508	Anaxyrus	punctatus
7460	1508	Gastrophryne	olivacea
7460	1508	Hyla	arenicolor
7460	1508	Incilius	alvarius
7460	1508	Lithobates	catesbeianus
7460	1508	Lithobates	chiricahuensis
7460	1508	Scaphiopus	couchii
7460	1508	Smilisca	fodiens
7460	1508	Spea	multiplicata
7550	1707	Anaxyrus	boreas
7550	1707	Ambystoma	gracile
7550	1707	Ambystoma	macrodactylum
7550	1707	Pseudacris	regilla
7550	1707	Rana	cascadae
7550	1707	Rana	pretiosa
7550	1707	Taricha	granulosa
7550	1709	Anaxyrus	boreas
7550	1709	Ambystoma	gracile
7550	1709	Ambystoma	macrodactylum
7550	1709	Pseudacris	regilla
7550	1709	Rana	cascadae
7550	1709	Rana	pretiosa
7550	1709	Taricha	granulosa
7550	1801	Anaxyrus	boreas
7550	1801	Ambystoma	gracile
7550	1801	Ambystoma	macrodactylum
7550	1801	Pseudacris	regilla
7550	1801	Rana	cascadae
7550	1801	Rana	pretiosa
7550	1801	Taricha	granulosa
7600	105	Ambystoma	maculatum
7600	105	Lithobates	sylvaticus
7610	109	Ambystoma	maculatum
7610	109	Lithobates	sylvaticus
7620	206	Ambystoma	maculatum
7620	206	Lithobates	sylvaticus
7630	107	Ambystoma	maculatum
7630	107	Lithobates	sylvaticus
7640	207	Ambystoma	maculatum
7640	207	Lithobates	sylvaticus

7650	106	Ambystoma	maculatum
7650	106	Lithobates	sylvaticus
7660	502	Ambystoma	maculatum
7660	502	Lithobates	sylvaticus
7670	501	Ambystoma	maculatum
7670	501	Lithobates	sylvaticus
7680	105	Ambystoma	maculatum
7680	105	Lithobates	sylvaticus
7690	203	Ambystoma	maculatum
7690	203	Lithobates	sylvaticus
7700	413	Ambystoma	maculatum
7700	413	Lithobates	sylvaticus
7710	207	Desmognathus	fuscus
7710	207	Eurycea	bislineata
7710	207	Pseudotriton	ruber
7720	207	Desmognathus	fuscus
7720	207	Eurycea	bislineata
7720	207	Pseudotriton	ruber
7720	207	Desmognathus	fuscus
7720	207	Eurycea	bislineata
7720	207	Pseudotriton	ruber
7740	705	Pseudacris	crucifer
7740	707	Pseudacris	crucifer
7750	902	Pseudacris	crucifer
7760	703	Pseudacris	crucifer
7770	704	Pseudacris	crucifer
7770	706	Pseudacris	crucifer
7800	1701	Anaxyrus	boreas
7800	1701	Ambystoma	macrodactylum
7800	1701	Rana	luteiventris
7800	1001	Anaxyrus	boreas
7800	1001	Ambystoma	macrodactylum
7800	1001	Rana	luteiventris
7800	1003	Anaxyrus	boreas
7800	1003	Ambystoma	macrodactylum
7800	1003	Rana	luteiventris
7800	1005	Anaxyrus	boreas
7800	1005	Ambystoma	macrodactylum
7800	1005	Rana	luteiventris
7811	1202	Acris	blanchardi
7811	1202	Gastrophryne	carolinensis
7811	1202	Hyla	cinerea
7811	1202	Incilius	nebulifer

7811	1202	Lithobates	catesbeianus
7811	1202	Lithobates	clamitans
7811	1202	Lithobates	sphenocephalus
7811	1202	Pseudacris	crucifer
7812	1202	Acris	blanchardi
7812	1202	Gastrophryne	carolinensis
7812	1202	Hyla	chrysoscelis
7812	1202	Hyla	cinerea
7812	1202	Incilius	nebulifer
7812	1202	Lithobates	catesbeianus
7812	1202	Lithobates	clamitans
7812	1202	Lithobates	sphenocephalus
7812	1202	Pseudacris	crucifer
7840	1807	Anaxyrus	californicus
7850	1807	Anaxyrus	boreas
7850	1807	Anaxyrus	californicus
7850	1807	Lithobates	catesbeianus
7850	1807	Pseudacris	cadaverina
7850	1807	Pseudacris	hypochondriaca
7860	1807	Anaxyrus	boreas
7860	1807	Anaxyrus	californicus
7860	1807	Lithobates	catesbeianus
7860	1807	Pseudacris	cadaverina
7860	1807	Pseudacris	hypochondriaca
7870	1807	Anaxyrus	boreas
7870	1807	Anaxyrus	californicus
7870	1807	Lithobates	catesbeianus
7870	1807	Pseudacris	cadaverina
7870	1807	Pseudacris	hypochondriaca
7880	1807	Anaxyrus	boreas
7880	1807	Anaxyrus	californicus
7880	1807	Lithobates	catesbeianus
7880	1807	Pseudacris	cadaverina
7880	1807	Pseudacris	hypochondriaca
7900	1704	Anaxyrus	boreas
7900	1704	Ambystoma	mavortium
7900	1704	Pseudacris	maculata
7900	1704	Rana	luteiventris
7900	1007	Anaxyrus	boreas
7900	1007	Ambystoma	mavortium
7900	1007	Pseudacris	maculata
7900	1007	Rana	luteiventris
7900	1002	Anaxyrus	boreas

7900	1002	Ambystoma	mavortium
7900	1002	Pseudacris	maculata
7900	1002	Rana	luteiventris
7920	710	Anaxyrus	americanus
7920	710	Hyla	versicolor/chrysoscelis
7920	710	Lithobates	catesbeianus
7920	710	Lithobates	pipiens
7920	710	Pseudacris	maculata
8100	1401	Anaxyrus	boreas
8100	1401	Ambystoma	mavortium
8100	1401	Lithobates	sylvaticus
8100	1401	Pseudacris	maculata
8100	1019	Anaxyrus	boreas
8100	1019	Ambystoma	mavortium
8100	1019	Lithobates	sylvaticus
8100	1019	Pseudacris	maculata
8101	1706	Ambystoma	macrodactylum
8101	1706	Rana	luteiventris
8170	208	Plethodon	cinereus
8170	208	Plethodon	shenandoah
8170	207	Plethodon	cinereus
8170	207	Plethodon	shenandoah
8180	207	Ambystoma	maculatum
8180	207	Lithobates	sylvaticus
8190	204	Ambystoma	maculatum
8190	204	Lithobates	sylvaticus
8200	202	Ambystoma	maculatum
8200	202	Lithobates	sylvaticus
9370	1807	Anaxyrus	boreas
9370	1807	Batrachoseps	major
9370	1807	Pseudacris	hypochondriaca
9370	1807	Spea	hammondii
9380	1807	Anaxyrus	boreas
9380	1807	Batrachoseps	major
9380	1807	Pseudacris	hypochondriaca
9380	1807	Spea	hammondii
9385	1807	Pseudacris	cadaverina
9385	1807	Rana	muscosa
9385	1810	Pseudacris	cadaverina
9385	1810	Rana	muscosa
9385	1809	Pseudacris	cadaverina
9385	1809	Rana	muscosa
9390	1807	Anaxyrus	boreas

9390	1807	Batrachoseps	species
9390	1807	Pseudacris	hypochondriaca
9390	1807	Ensatina	eschscholtzii
9390	1807	Batrachoseps	major
9390	1807	Aneides	lugubris
9390	1807	Spea	hammondii
9395	1807	Anaxyrus	boreas
9395	1807	Batrachoseps	major
9395	1807	Pseudacris	hypochondriaca
9395	1807	Aneides	lugubris
9395	1807	Batrachoseps	species
9395	1807	Spea	hammondii
9395	1807	Ensatina	eschscholtzii

Supplementary Information Table S3: Genus and species for codes used in Figure 2.

spnum	genus	sp
A01	Anaxyrus	cognatus
A02	Anaxyrus	punctatus
A03	Gastrophryne	olivacea
A04	Hyla	arenicolor
A05	Incilius	alvarius
A06	Lithobates	chiricahuensis
A07	Scaphiopus	couchii
A08	Smilisca	fodiens
A09	Spea	multiplicata
A10	Rana	luteiventris
A11	Rana	aurora
A12	Lithobates	grylio
A13	Hyla	squirella
A14	Pseudacris	fouquettei
A15	Anaxyrus	fowleri
A16	Acris	blanchardi
A17	Incilius	nebulifer
A18	Hyla	cinerea
A19	Hyla	chrysoscelis
A20	Gastrophryne	carolinensis
A21	Anaxyrus	woodhousii
A22	Hyla	versicolor
A23	Incilius	nebulifer
A24	Pseudacris	triseriata
A25	Acris	crepitans
A26	Acris	gryllus
A27	Anaxyrus	terrestris
A28	Pseudacris	feriarum
A29	Pseudacris	nigrita
A30	Pseudacris	ocularis
A31	Pseudacris	ornata
A32	Rana	cascadae
A33	Rana	pretiosa
A34	Pseudacris	regilla
A35	Rana	draytonii
A36	Lithobates	sphenocephalus
A37	Anaxyrus	californicus
A38	Pseudacris	hypochondriaca
A39	Spea	hammondii
A40	Pseudacris	cadaverina
A41	Lithobates	catesbeianus
A42	Anaxyrus	boreas
A43	Anaxyrus	canorus

A44	Rana	sierrae
A45	Rana	muscosa
A46	Lithobates	clamitans
A47	Anaxyrus	woodhousii
A48	Lithobates	palustris
A49	Lithobates	septentrionalis
A50	Lithobates	sylvaticus
A51	Pseudacris	crucifer
A52	Lithobates	pipiens
A53	Hyla	versicolor/chrysoscelis
A54	Anaxyrus	fowleri/americanus
A55	Anaxyrus	americanus
A56	Pseudacris	maculata

85

C01	Ambystoma	mavortium
C02	Aneides	lugubris
C03	Plethodon	glutinosus
C04	Plethodon	jordani
C05	Plethodon	serratus
C06	Desmognathus	fuscus
C07	Ambystoma	maculatum
C08	Pseudotriton	ruber
C09	Plethodon	cinereus
C10	Ambystoma	gracile
C11	Taricha	granulosa
C12	Eurycea	cirrigera
C13	Eurycea	guttolineata
C14	Gyrinophilus	porphyriticus
C15	Ambystoma	talpoideum
C16	Ambystoma	bishopi
C17	Ambystoma	californiense
C18	Ambystoma	macrodactylum
C19	Taricha	torosa
C20	Batrachoseps	species
C21	Ensatina	eschscholtzii
C22	Desmognathus	imitator
C23	Desmognathus	wrighti
C24	Eurycea	wilderae
C25	Plethodon	shenandoah
C26	Ambystoma	opacum
C27	Eurycea	bislineata
C28	Hemidactylium	scutatum
C29	Notophthalmus	viridescens

```
#### Example BUGS code for implementation of occupancy model including Bayesian LASSO  ####
for (i in 1:nobs){
  z[i] ~ dbern(psi[groupyear[i]])  ### latent occupancy state
        P[i] <- p[groupyear[i]]*z[i]     ### probability of observing species during a visit
        D[i] ~ dbin(P[i],N[i])          ### data model - D is observed number of detections  N is number of visits
}
for (j in 1:ngroup){
        alpha[j] ~ dnorm(mualpha,taualpha)  ## group specific random effect
}

for (k in 1:ngroupyear){
   ###  occupancy model - function of group specific random effect, beta parameters and covariates, and annual observation error term
  logit(psi[k]) <-  alpha[group[k]] + s1[k]*b[1] + s2[k]*b[2] + s3[k]*b[3] + s4[k]*b[4] + s5[k]*b[5] + s6[k]*b[6] + s7[k]*b[7] + s8[k]*b[8] + s9[k]*b[9] + obserr[k]#
   ### detection parameters
  det[k] ~ dnorm(mup,taup)
        logit(p[k]) <- det[k]
   ### annual error term - unexplained variation from the long-term trend
  obserr[k] ~ dnorm(0,tauerr)
}

## priors for intercept terms (uniform on real scale)
mp ~ dunif(0,1)
malpha ~ dunif(0,1)
mup <-  log(mp) - log(1-mp)
mualpha <-  log(malpha) - log(1-malpha)

#####  priors for beta parameters with Bayesian lasso (double-exponential prior)
for (a in 1:9){
  b[a] ~ dnorm(0,tauB)
}

B ~ dnorm(0,0.01)
tauB <- pow(sig2B,-1)
sig2B ~ dexp(lambda2)
lambda2 <- pow(lambda,2)
lambda ~ dunif(0,100)

#### additional priors for random-effect parameters
taup <- pow(sigp,-2)
taualpha <- pow(sigalpha,-2)
tauerr <- pow(sigerr,-2)
sigp ~ dunif(0,3)
sigalpha ~ dunif(0,3)
sigerr ~ dunif(0,3)
```
